# Supplementary figures and images for: The impact of sanctuary visits on children’s knowledge and attitudes toward primate welfare and conservation
Source: PeerJ. 2023 Jun 16;11:e15074. doi: 10.7717/peerj.15074 (PMC10284066; doi:10.7717/peerj.15074)

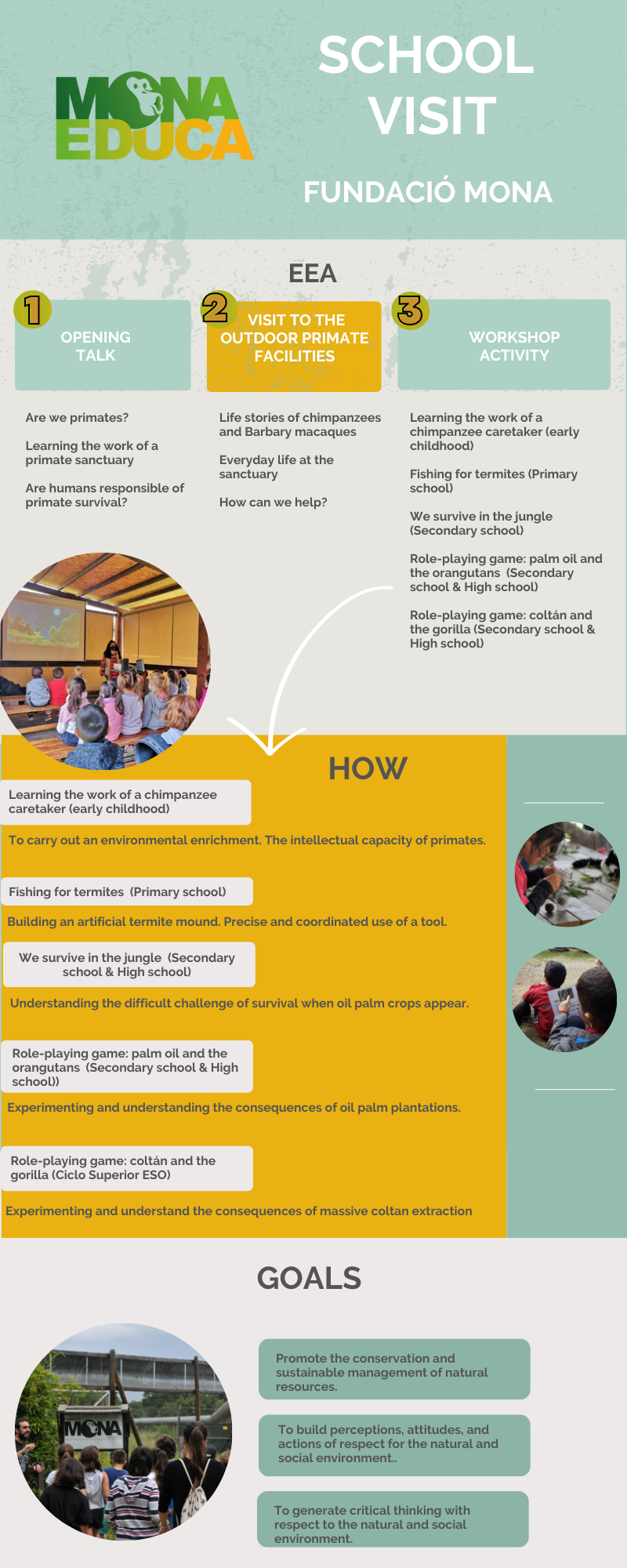

Supplement: Supplemental Information 1 [file peerj-11-15074-s001.png]
