# Supplementary material for: The impact of sanctuary visits on children’s knowledge and attitudes toward primate welfare and conservation
Source: PeerJ. 2023 Jun 16;11:e15074. doi: 10.7717/peerj.15074 (PMC10284066; doi:10.7717/peerj.15074)
Supplement: Supplemental Information 4 — A) Results of the linear model for general impact of Knowledge. B) Results of the linear mixed model for general impact of Knowledge for category. C) Results of the linear model for general impact of attitudes. [file peerj-11-15074-s004.pdf]

Supplementary Material.

**V.I**	***F***	**df**	***P* value**
EEA	788.520	1	**0.00****
Grade	11.72	2	**0.00****
Sex	0.7721	1	0.533

Supplementary Table. 1. Results of the linear model for general impact of Knowledge. *F* corresponds to the F-statistic. df to the degrees freedom. *P* to the P Value where * is a significance of < 0.5, ** is a significance of < 0.01.

**V..I**	***F***	**df**	***P* value**
Category	135.520	1	**0.00****
Sex	4.45	1	**0.01***
Sex x Category	11.007	1	**0.01***

Supplementary Table 2. Results of the linear mixed model for general impact of Knowledge for Category.. *F* corresponds to the F-statistic. df to the degrees freedom. *P* to the P Value where * is a significance of < 0.5, ** is a significance of < 0.01.

**V..I**	***F***	**df**	***P* value**
EEA	347.283	1	**0.00****
Grade	0.239	2	0.787
Sex	34.249	1	**0.00****

Supplementary Table 3. Results of the linear model for general impact of attitudes. *F* corresponds to the F-statistic. df to the degrees freedom. *P* to the P Value where * is a significance of < 0.5, ** is a significance of < 0.01.
