# Supplementary material for: The impact of sanctuary visits on children’s knowledge and attitudes toward primate welfare and conservation
Source: PeerJ. 2023 Jun 16;11:e15074. doi: 10.7717/peerj.15074 (PMC10284066; doi:10.7717/peerj.15074)
Supplement: Supplemental Information 5 [file peerj-11-15074-s005.pdf]

ESCOLA CONEIXEMENT PRE

L’ any 2004 la Fundació MONA va iniciar el seu programa educatiu. Des de llavors ens han visitat moltes persones que han après aspectes nous dels primats i han conegut la seva realitat. Ara volem recollir aquestes dades de manera més formal per tal de valorar el programa educatiu amb el qual treballem.
Agrairíem contestessis les preguntes d’ aquest qüestionari. Ens ajudarà molt a seguir millorant.

Gràcies per la teva col·laboració.

1. Data

____

Exemple: 7 de gener de 2019

2. Edat

____

3. Curs

Marqueu només un oval.

- ( ) 3er
- ( ) 4rt
- ( ) 5è
- ( ) 6è
- ( ) 1er o 2on ESSO
- ( ) 3er o 4rt ESSO
- ( ) Batxillerat

4. Marca el que correspongui

Marqueu només un oval.

- Nen
- Nena

5. Si poguessis tenir un ximpanzé a casa des de petit creus que podria ser una bona mascota.

Marqueu només un oval.

- Si, totalment d' acord
- Depen del carácter del ximpanzé
- No. Mai

6. El ximpanzé és un animal:

Marqueu només un oval.

- solitari / viu sol
- social /viu en grup

7. Creus que els ximpanzés son bons per fer anuncis a la tele?

Marqueu només un oval.

- Si
- No

8. Entrenar animals com ximpanzés perquè surtin en pel·lícules o anuncis els perjudica?

Marqueu només un oval.

- Si
- No, Si els tracten bé.

9. Els ximpanzés

Marqueu només un oval.

- Són animals que estan en perill d' extinció
- Són una espècie vulnerable
- No estan en perill. Encara n' hi han molts

10. Quants anys pot viure un ximpanzé en captivitat?

Marqueu només un oval.

- 10-15 anys
- 20-30 anys
- 50-60 anys
- 90-100 anys

11. Quant pot arribar a pesar un ximpanzé mascle adult?

Marqueu només un oval.

- 20-30 kg
- 50-60 kg
- 70-80 kg
- Més de 90Kg

12. Un centre de recuperació de primats com la Fundación MONA es dedica a:

Marqueu només un oval.

- Recuperar i socialitzar primats que provenen de ser mascotes o d' actuar en circs i televisió
- Cuidar primats fins que estiguin bé i després tornar-los a la selva
- Guarir primats que han estat ferits a la selva
- Totes les anteriors són correctes

Google no ha creat ni aprovat aquest contingut.

Google Formularis
