# Supplementary material for: The impact of sanctuary visits on children’s knowledge and attitudes toward primate welfare and conservation
Source: PeerJ. 2023 Jun 16;11:e15074. doi: 10.7717/peerj.15074 (PMC10284066; doi:10.7717/peerj.15074)
Supplement: Supplemental Information 6 [file peerj-11-15074-s006.pdf]

ESCOLA ACTITUD PRE

L' objectiu d' aquest formulari és fer una valoració de les activitats del programa educatiu de la Fundació MONA i comprovar si compleixen l' objectiu pel qual es van crear. Agrairíem contestéssiu totes les preguntes.

Aquestes preguntes van associades a les imatges que us lliurem.

Gràcies per la teva col.laboració.

1. Data

____

Exemple: 7 de gener de 2019

2. Edat

____

3. Curs

Marqueu només un oval.

- ( ) 3er
- ( ) 4rt
- ( ) 5è
- ( ) 6è
- ( ) 1er o 2on ESSO
- ( ) 3er o 4rt ESSO
- ( ) BATXILLERAT

4. Marca el que correspongui

Marqueu només un oval.

- ( ) Nen
- ( ) Nena

5. 1. A quin grup creus que pertanyen els ximpanzés?

Marqueu només un oval.

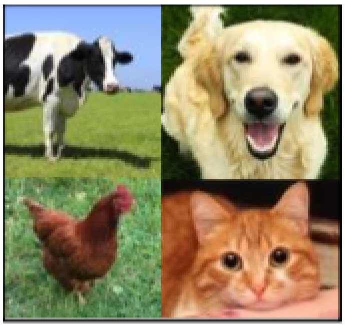

b)

6. 2. Quina d'aquestes fotos prefereixes veure en un anunci de Fundació Mona?

Marqueu només un oval.

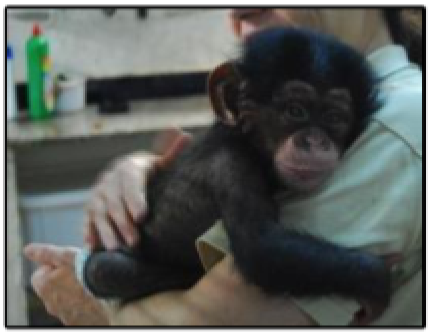

a)

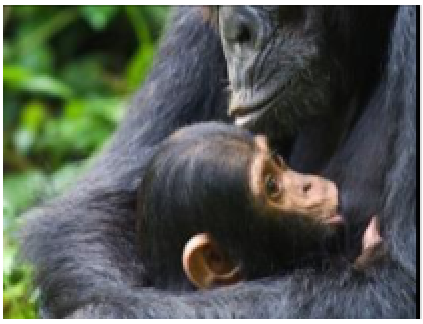

b)

7. 3. A quin grup creus que pertanyen els ximpanzés?

Marqueu només un oval.

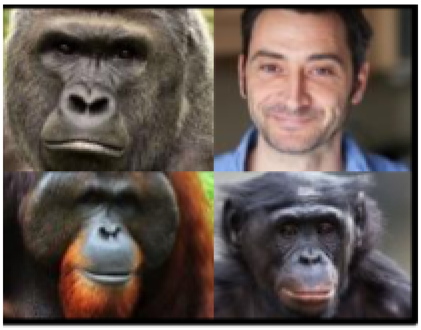

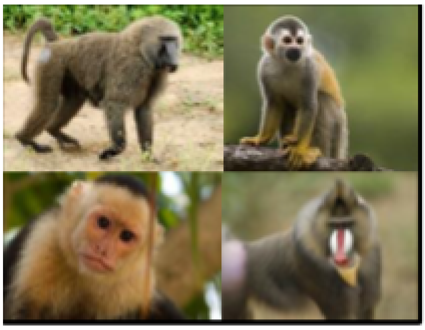

- ( ) a)
- ( ) b)

8. 4. Com t' agradaria més veure el bosc?

Marqueu només un oval.

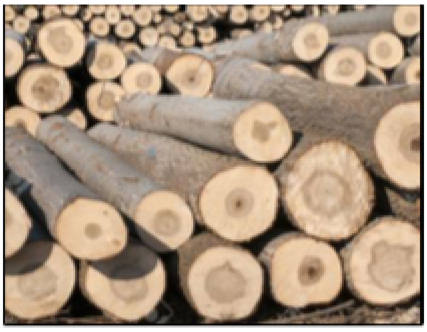

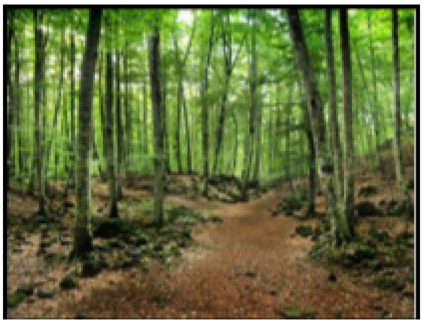

- ( ) a)
- ( ) b)

9. 5. A quin grup creus que pertanyen els ximpanzés?

Marqueu només un oval.

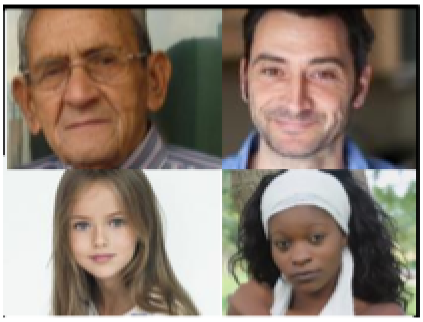

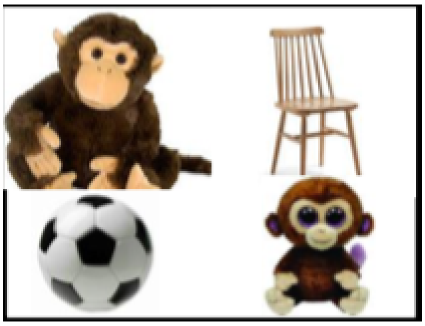

a)

b)

10. 6. Quina d'aquestes fotos prefereixes veure en un anunci de Fundació Mona?

Marqueu només un oval.

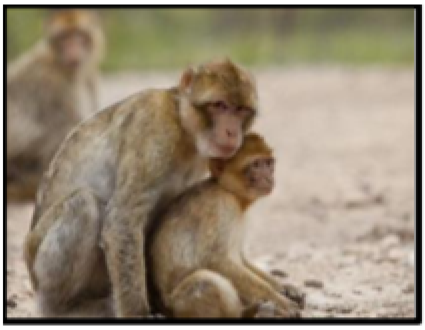

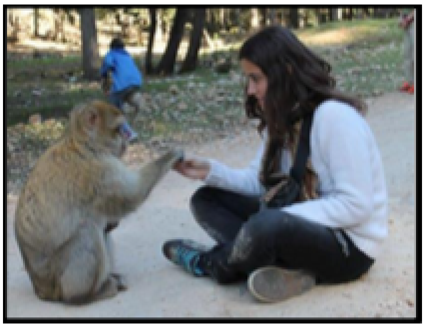

a)

b)

11. 7. En quina d’aquestes dues situacions preferiries estar?

Marqueu només un oval.

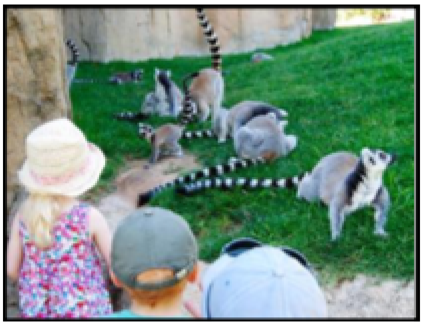

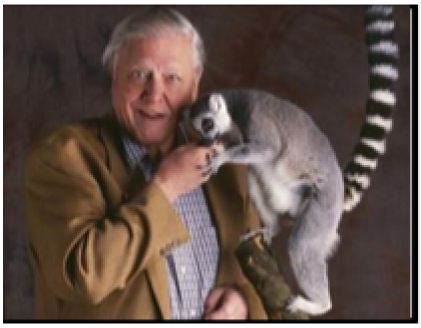

( ) a) ( ) b)

12. 8. Com t’agrada més veure a aquest ximpanzé?

Marqueu només un oval.

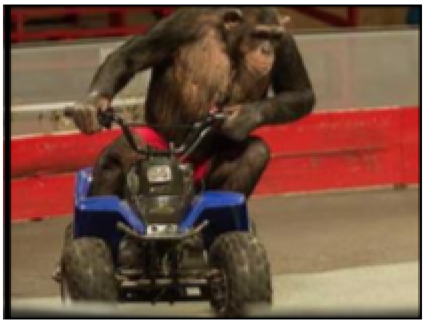

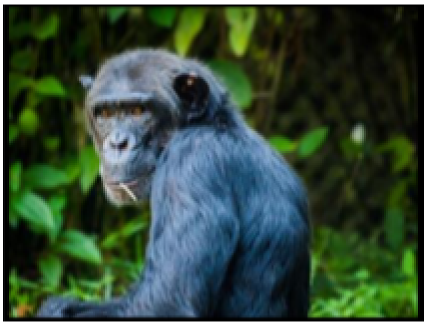

( ) a) ( ) b)

Google no ha creat ni aprovat aquest contingut.

Google Formularis
