# Supplementary material for: The impact of sanctuary visits on children’s knowledge and attitudes toward primate welfare and conservation
Source: PeerJ. 2023 Jun 16;11:e15074. doi: 10.7717/peerj.15074 (PMC10284066; doi:10.7717/peerj.15074)
Supplement: Supplemental Information 7 [file peerj-11-15074-s007.docx]

**Knowledge Questionnaire**

Date:

Age:

Grade:

Choose what applies: Boy

Girl

Q1: If you can have a chimpanzee at home since being a baby, do you think it could be a good pet?

- Yes, I completely agree
- It depends on the character of the chimpanzee
- No. Never

Q2: The chimpanzee is

- A solitary animal/ He lives on his own
- A social animal/ He lives in groups

Q3: Do you think chimpanzees are good for TV commercials?

- Yes
- No

Q4: Does training animals like chimpanzees to participate in movies or commercials hurt them?

- Yes
- No. If they are well treated

Q5: The chimpanzees

- Are endangered animals
- Are vulnerable animals
- Are not threatened. There are many left.

Q6: How many years a chimpanzee can live in captivity?

- 10 – 15 years
- 20 – 30 years
- 50 – 60 years
- 90 – 100 years

Q7: How much can an adult male chimpanzee weight?

- 20 – 30 Kg
- 50 – 60 Kg
- 70 – 80 Kg
- More than 90 Kg

Q8: A primate rescue center like the Mona sanctuary

- Rescues and socializes primates that come from circuses, TV commercials and the pet trade
- Heals the chimpanzees and then takes them back to their habitat
- Heals primates that have been injured in the jungle
- All of these are correct
