# Supplementary material for: The impact of sanctuary visits on children’s knowledge and attitudes toward primate welfare and conservation
Source: PeerJ. 2023 Jun 16;11:e15074. doi: 10.7717/peerj.15074 (PMC10284066; doi:10.7717/peerj.15074)

**Attitudes Questionnaire**

Date:

Age:

Grade:

Choose what applies: Boy

Girl

Q1: Which group do you think chimpanzees belong to?


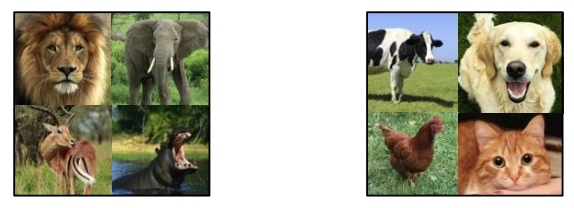


Q2: Which of these photos do you prefer to see in a Mona sanctuary ad?


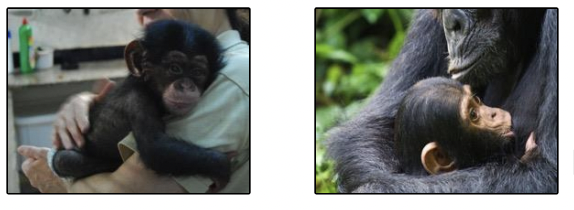


Q3: Which group do you think chimpanzees belong to?


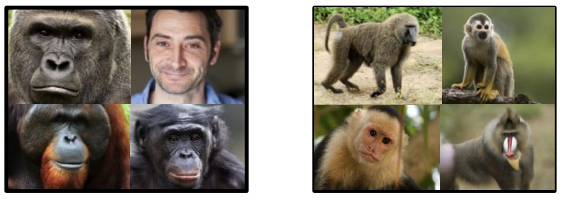


Q4: Which photo best shows the value of the forest?


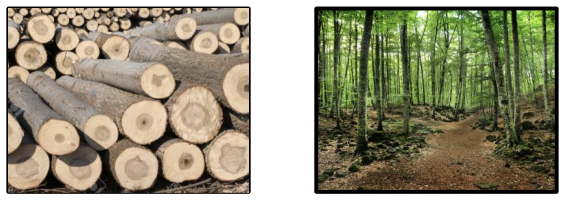


Q5: Which group do you think chimpanzees belong to?


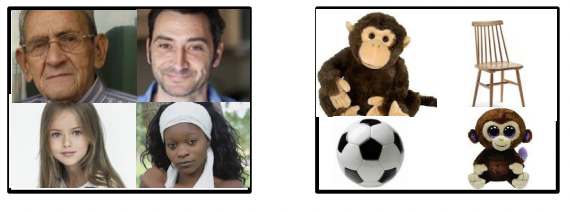


Q6: Which of these photos do you prefer to see in a Mona sanctuary ad?


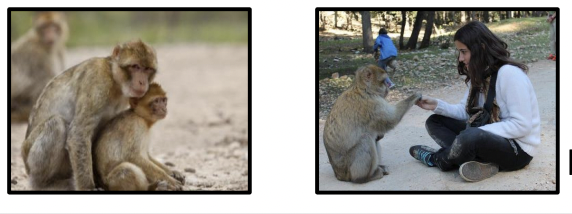


Q7: Which of these two situations do you prefer to be in?


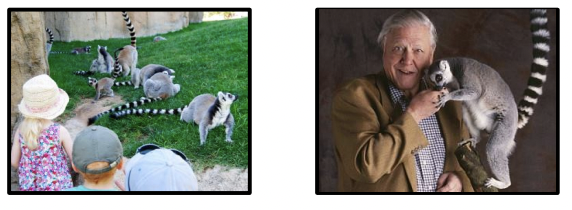


Q8: How do you like to see this chimpanzee?


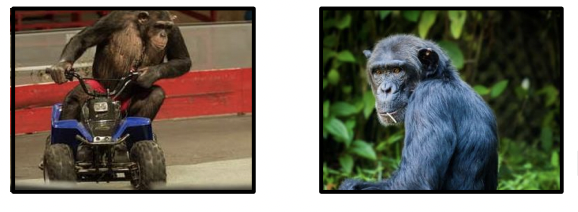

Supplement: Supplemental Information 8 [file peerj-11-15074-s008.docx]
